# Supplementary material for: Flatfoot in Africa, the cirripede Chthamalus in the west Indian Ocean
Source: PeerJ. 2021 Jul 8;9:e11710. doi: 10.7717/peerj.11710 (PMC8272926; doi:10.7717/peerj.11710)
Supplement: Supplemental Information 1 [file peerj-09-11710-s001.docx]

Simon-Blecher et al. Flatfoot in Africa: The cirripede *Chthamalus* in the west Indian Ocean.

Supplementary material 1: Material studied

Samples studied are deposited at the Israeli National Natural History Collections at the Hebrew University of Jerusalem

***Chthamalus barilani***

HUJIVRCRUSCIRR225 *Chthamalus barilani* Morondava*,* Madagasca*r,* 21. June 2019. Holotype wet sample

HUJIVRCRUSCIRR226 *Chthamalus barilani, Nosy be, Madagascar,* 21. June 2019. sample 1 *Paratype Dry sample*

HUJIVRCRUSCIRR227 *Chthamalus barilani Nosy be, Madagascar,* 21. June 2019. sample 1 *Paratype Dry sample*

HUJIVRCRUSCIRR234 *Chthamalus barilani,* Morondava*,* Madagascar *,* 21. June 2019. Holotype wet sample 1 wet sample, lot

HUJIVRCRUSCIRR229: *Chthamalus barilani,* Morondava, Madagascar *,* 21. June 2019. SEM stabs, opercular valves

HUJIVRCRUSCIRR230: *Chthamalus barilani,* Nosy Be, Madagascar *,* 27. June 2019. SEM stabs, opercular valves

HUJIVRCRUSCIRR231: *Chthamalus barilani,* Belo sur Mer Madagascar, 23. June 2019 SEM stabs, opercular valves

HUJIVRCRUSCIRR232: *Chthamalus barilani, Chthamalus malayensis.* Zanzibar mangroves. 7.7. 2016

HUJIVRCRUSCIRR233: *Chthamalus barilani, Chthamalus malayensis,* Zanzibar, mangroves. 7.7. 2016 (Ethanol)

HUJIVRCRUSCIRR234: *Chthamalus barilani,* Nosy Be, Madagascar *,* 27. June 2019. SEM stabs, gold coated Artropodal appendages

HUJIVRCRUSCIRR235: *Chthamalus barilani,* Nosy Be, Madagascar*,* 27. June 2019. SEM stabs, gold coated Shell and opercular valves.

HUJIVRCRUSCIRR236: *Chthamalus barilani,* Belo sur Mer, Madagascar*,* 30. June 2019. SEM stabs, dry Shells.

HUJIVRCRUSCIRR237: *Chthamalus barilani,* Nosy Be, Madagascar*,* 30. June 2019. SEM stabs, dry shell.

HUJIVRCRUSCIRR238: *Chthamalus barilani,* Morondava, Madagascar*,* 27. June 2019. SEM stabs, dry shells.

HUJIVRCRUSCIRR239 *Chthamalus barilani,* Morondava*,* Madagascar *,* 21. June 2019. Microscopic slides

HUJIVRCRUSCIRR240 *Chthamalus barilani,* Nosy be*,* Madagascar *,* 21. June 2019. Microscopic slides

HUJIVRCRUSCIRR241 *Chthamalus barilani,* Belo sur Mer*,* Madagascar *,* 21. June 2019. Microscopic slides

HUJIVRCRUSCIRR242 *Chthamalus barilani,* Belo sur Mer*,* Madagascar *,* 21. June 2019. SEM stabs, spats

***Chthamalus malayensis***

HUJIVRCRUSCIRR251: *Chthamalus malayensis,* Zanzibar, Turtle Island, 6.7.2016 (Dry)

HUJIVRCRUSCIRR252: *Chthamalus spp*. Dar e Salaam, Tanzania, microscopic slides

HUJIVRCRUSCIRR253: *Chthamalus malayensis,* Zanzibar, Turtle Island, 6.7.2016 Opercular valves Gold coated removed from SEM stabs. (Dry)

HUJIVRCRUSCIRR254: *Chthamalus malayensis,* Zanzibar, Turtle Island, 6.7.2016 in Ethanol

HUJIVRCRUSCIRR255: *Chthamalus malayensis,* Dar e Salaam, Tanzania. June.2018 in Ethanol.

HUJIVRCRUSCIRR256: *Chthamalus malayensis,* Dar e Salaam, Tanzania. June.2018 Fixed in ethanol dried

HUJIVRCRUSCIRR257: *Chthamalus malayensis*, Dar e Salaam, Tanzania, Zanzibar Turtle Island. microscopic slides

HUJIVRCRUSCIRR258: *Chthamalus malayensis*, Dar e Salaam, Tanzania Island. Dry, gold coated on SEM stabs

HUJIVRCRUSCIRR259: *Chthamalus malayensis*, Zanzibar Turtle is. and Dar e Salaam, Tanzania Island. Microscopic slides

***Chthamalus dentatus***

HUJIVRCRUSCIRR261: *Chthamalus dentatus,* Morondava*,* Madagascar *,* 21. June 2019 lot in ethanol.

HUJIVRCRUSCIRR262: *Chthamalus dentatus,* Morondava*,* Madagascar *,* 21. June 2019 lot dry sample, (two gold coated for SEM study)

HUJIVRCRUSCIRR263: *Chthamalus dentatus,* Morondava*,* Madagascar *,* 21. June 2019 lot dry sample, gold coated for SEM study.

HUJIVRCRUSCIRR264: *Chthamalus dentatus,* Morondava*,* Madagascar *,* 21. June 2019 lot dry sample (4 specimens), opercular valves gold coated for SEM study, shells dry.

***Chthamalus barnesi***

HUJIVRCRUSCIRR265: *Chthamalus barnes, i* Elat, Israel:

HUJIVRCRUSCIRR266: *Chthamalus barnesi,* Gulf of Oman Coll Anonymous:

HUJIVRCRUSCIRR267: *Chthamalus barnesi,* Yeman, Coll Anonymous

HUJIVRCRUSCIRR268a: *Chthamalus barnesi,* Hormoz Is. Persian Gulf: Coll Anonymous In ethanol

HUJIVRCRUSCIRR268b: *Chthamalus barnes,* Hurmuz Is. Persian Gulf: Coll Anonymous Fixed in ethano, Dried.

HUJIVRCRUSCIRR270: *Chthamalus barnesi,* Persian Gulf: in ethanol Coll Anonymous

HUJIVRCRUSCIRR64: *Chthamalus barnesi,* Nabeq, Egypt: In Ethanol

HUJIVRCRUSCIRR116: *Chthamalus barnesi*, Ras-Misela, Egypr 18 Feb 1969 in ethanol
